# Time to exploit phenotypic plasticity

**DOI:** 10.1093/jxb/eraa268

**Published:** 2020-09-19

**Authors:** Antonio J Monforte

**Affiliations:** Instituto de Biología Molecular y Celular de Plantas (IBMCP), Universitat Politècnica de València (UPV)-Consejo Superior de Investigaciones Científicas (CSIC), Valencia, Spain

**Keywords:** Adaptation, breeding, climate change, genotype×environment, multiple stress

## Abstract

This article comments on:

**Diouf I, Derivot L, Koussevitzky S, Carretero Y, Bitton F, Moreau L, Causse M**. 2020. Genetic basis of phenotypic plasticity and genotype×environment interaction in a multi-parental tomato population. Journal of Experimental Botany **71**, 5365–5376.

**The study of plant phenotypic plasticity complements our knowledge of plant response to stresses obtained from controlled single and multiple stress experiments. Diouf *et al.* (2020) dissect the genetic control of phenotypic plasticity for several traits in tomato. A few loci control both plasticity and mean phenotypes, while most loci are associated only with plasticity or mean phenotypes. The results can be applied to develop new cultivars for different objectives, from stable behavior to those specifically-adapted to different environments, by combining loci with different contributions to plasticity or mean phenotype.**

Land plants in natural environments have to respond to different stresses—including heat, cold, salinity, drought, metal toxicity, pests, and diseases, among others—to complete their life cycle. Defense responses for those stresses have evolved in all plant species. These include changes in cuticle (shield), unsaturated fatty acids (membrane modulator), reactive species scavengers (reactive species homeostasis), molecular chaperones (stabilize proteins and subcellular structures), compatible solutes (osmoprotectants), and cellular responses to pathogen attack (Cui *et al.*, 2015; He *et al.*, 2018). Selection pressure has maintained these robust defense responses in natural populations. Domestication of wild species changed the selection pressure towards human needs, and the development of agronomic management techniques reduced the need for adaptation to stressful environments. Modern breeding accelerated the sensitivity of cultivars to stresses by developing high-yielding cultivars with high-input requirements. Biotic and abiotic stress has always been a threat to agricultural production, but the threat is heightened in the current context of climate change, with more frequent extreme heat and drought events (IPPC, 2014; Wu *et al.*, 2018). Furthermore, population growth demands greater agriculture production with minimum ecological impact. The development of high-yielding cultivars capable of responding to changing environmental stresses is today one of the major goals for breeders.

## Plant response to abiotic stresses and breeding

Over the past years, an impressive body of knowledge has been generated by the scientific community on plant responses to stresses. The sequences of a large number of genes involved in tolerance to abiotic stresses have been reported (e.g. Gerszberg *et al.*, 2017; Ganie *et al.*, 2019) as well as natural variation and quantitative trait loci (QTLs; Ayean *et al*., 2019; Morton *et al.*, 2019). Most of these works applied the common scientific reductionist approach: they focused on one single stress and studied the plant response among limited stress levels based on historical values (Arnold *et al.*, 2019). However, combinations of two or more stresses (drought and heat or salinity, for example) are common in many agricultural areas around the world (Suzuki *et al.*, 2014). The effects of combinations of stresses are not additive; for example, combinations of drought with salinity, heat, chilling, pathogens, UV, nutrients, and heavy metals increase the negative effects of each individual stress, while combinations of drought with ozone or high CO_2_ mitigate the effect of the single stress (Suzuki *et al.*, 2014). Each stress combination imposes specific requirements on the plant and therefore different responses are found when comparing single and multiple stress responses (Zandalinas *et al.*, 2018). Thus, all the previous knowledge should be re-evaluated under multiple stress conditions. Moreover, the shape of the phenotypic response to certain stresses is not linear for most phenotypic traits; in fact, it is typically curved (Arnold *et al.*, 2019), which means it is also appropriate to reconsider conclusions based on limited stress levels. The limitations of common experimental designs may explain the difficulties in extrapolating the research results to applied breeding.

## Plasticity

Phenotypic plasticity is defined as the ability of a genotype to display different phenotypes as a response to different environments (Kusmec *et al.*, 2018). The degree of plasticity may vary from zero (phenotype is stable) to far from zero (phenotype is plastic). Variation in plasticity among genotypes is classically known as genotype×environment interaction (Box 1). From the applied point of view, the appropriate degree of plasticity depends on the expected environmental conditions. Reduced plasticity is selected for producing stable yields when environments are relatively homogeneous (e.g. intensive agriculture or greenhouses), although adaptation to future environments would be constrained due the genetic homogeneity of the selected cultivars (Gage *et al.*, 2017). High plasticity is needed to obtain cultivars adapted to specific environments, although lower yields would be obtained in non-target environments (Bernardo, 2010). Climate change forecasts predict that unstable weather will become more frequent, so high-yielding cultivars that provide predictable yields in a broader range of environments are one of the most important challenges facing breeders (Kusmec *et al.*, 2018). In order to exploit the plasticity in breeding programs, genetic control should be elucidated or environments developed for testing in a manner that maximizes the opportunity to identify plastic genotypes. The study of plasticity needs to include a wide range of environments, an appropriate mapping population, and powerful statistical frameworks (Arnold *et al.*, 2019). A few previous works have demonstrated that variation in plasticity has a genetic basis and is therefore amenable to selection (Mangin *et al*., 2017).

**Box 1. Differences in phenotypic plasticity among genotypes.**

Several hypothetical traits have been recorded in some genotypes across different environments. Genotypes showed different phenotypes for trait 1, but no differences among environments: no plasticity. For trait 2, genotypes responded to the different environments, but the response is similar in all genotypes: plasticity. Finally, for trait 3, the phenotypic response across environments is different among genotypes: variability in plasticity, genotype×environment interaction.

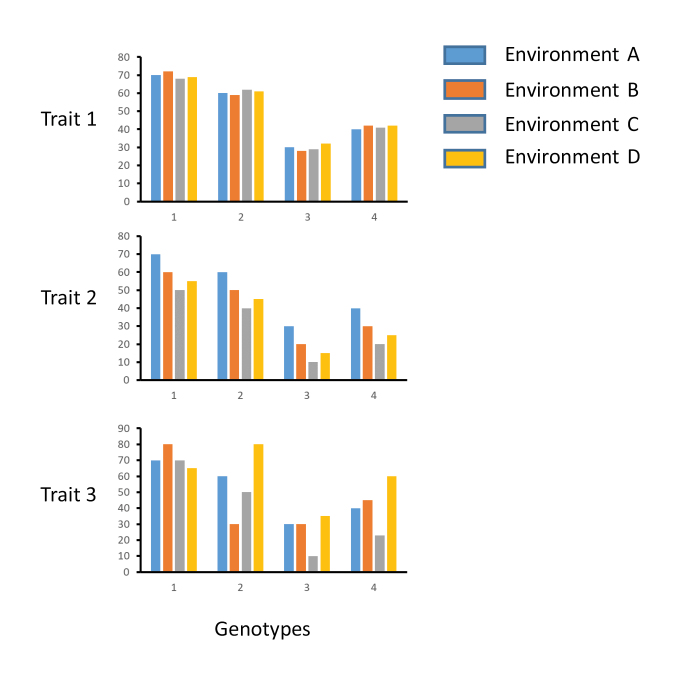

Diouf *et al.* (2020) used a multiparental advanced intercross (MAGIC) population, developed from intercrossing between eight tomato genotypes (Pascual *et al.*, 2015) to investigate plasticity for several phenological and agronomic traits. The MAGIC population was evaluated in 12 environments in three different experimental stations, with different levels of heat, water, and salinity. Each combination of experiment and stress treatment could be considered as a single environment in order to allow a simultaneous study of the impact of a single stress, or they could all be studied together for a suitable estimation of the general plasticity accounting for the variations between the different environments. The choice of a MAGIC population deserves to be highlighted. The genetic structure of MAGIC populations offers a good compromise between genetic variability (up to eight alleles may segregate) and balanced allele frequency (a minimum of 0.125 per allele), compared with biparental populations (two alleles) or genome-wide association panels (where rare alleles are common and their effects cannot be detected). The authors observed that the best average performing genotypes were usually the most plastic in their response. This association between higher plasticity and better agronomic performance was also observed by Mangin *et al.* (2017). Diouf *et al.* (2020) identified QTLs associated with phenotype means and plasticity. Twenty-one percent of them were involved in both phenotype mean and plasticity, which would explain the association between plasticity and agronomic performance. Interestingly, a hub of plasticity QTLs for several traits were identified on chromosome 11. Most plasticity QTLs were located within domestication and improvement sweep regions previously defined by Zhu *et al.* (2018), suggesting that selection by breeding of plasticity alleles confers good adaptability in high-quality environments. Their analysis also allowed them to obtain insight into the underlying genetic model (i.e. overdominance, allelic sensitivity, or gene regulatory) that better fits the plasticity in the current research. Given the homozygosity of the tested MAGIC lines, overdominance can be ignored. Of the other two genetic models, the gene regulatory model seems most reasonable because a large proportion of plasticity QTLs did not co-localize with main effect QTLs.

## Perspectives

We are changing our paradigm for studying crop responses to stresses and designing new crop varieties for future unpredictable environments. Powerful mapping populations, extensive trials, high-throughput phenotyping, new statistical models (Arnold *et al.*, 2019), and the inclusion of biotic stresses will allow the next generation of breeders to obtain a better understanding of the genetic control of plasticity and its exploitation in breeding programs. Diouf *et al.* (2020) and previous pioneering works (Gage *et al.*, 2017; Kusmec *et al.*, 2018) have shown that genetic main effects and plasticity have both common and independent genetic control. The determination of these common and specific loci will allow the design of more efficient breeding strategies for different objectives, from varieties adapted to local conditions to varieties with high-yield stability over contrasting environments. The identification of the causal genes underlying plasticity will help us to better understand the complex responses of plants to stresses.
